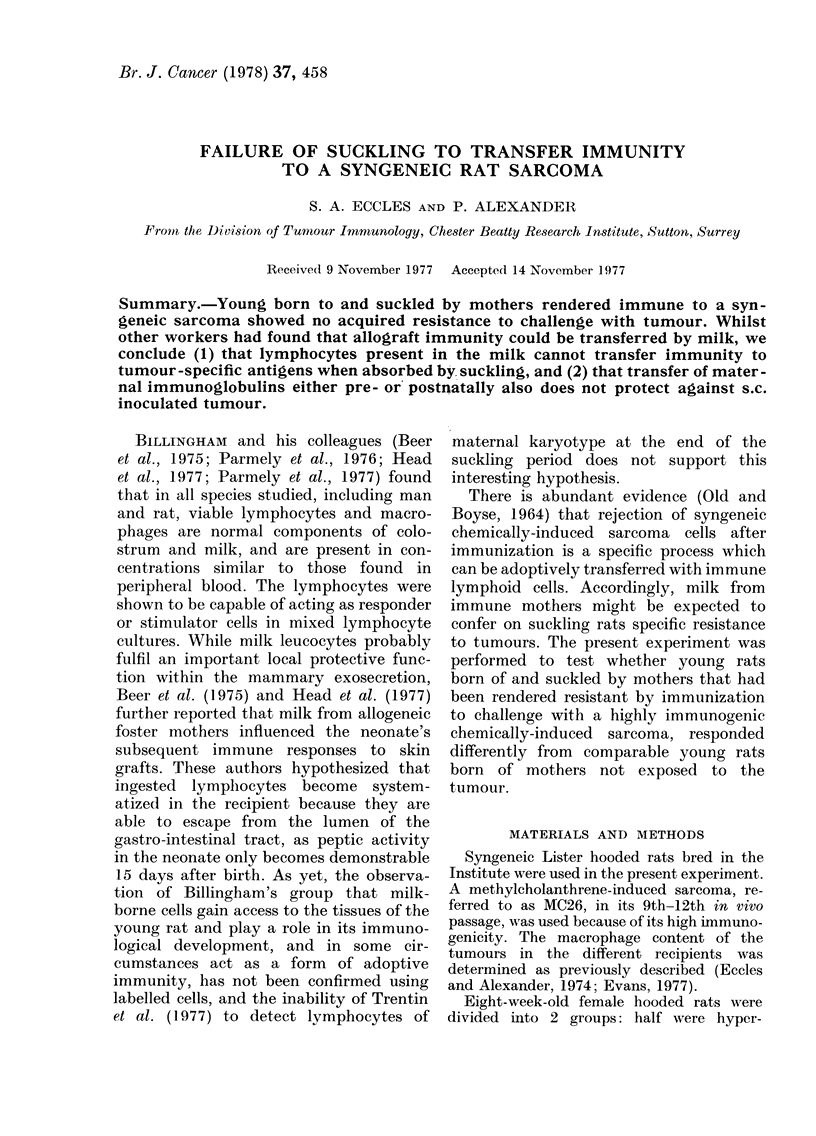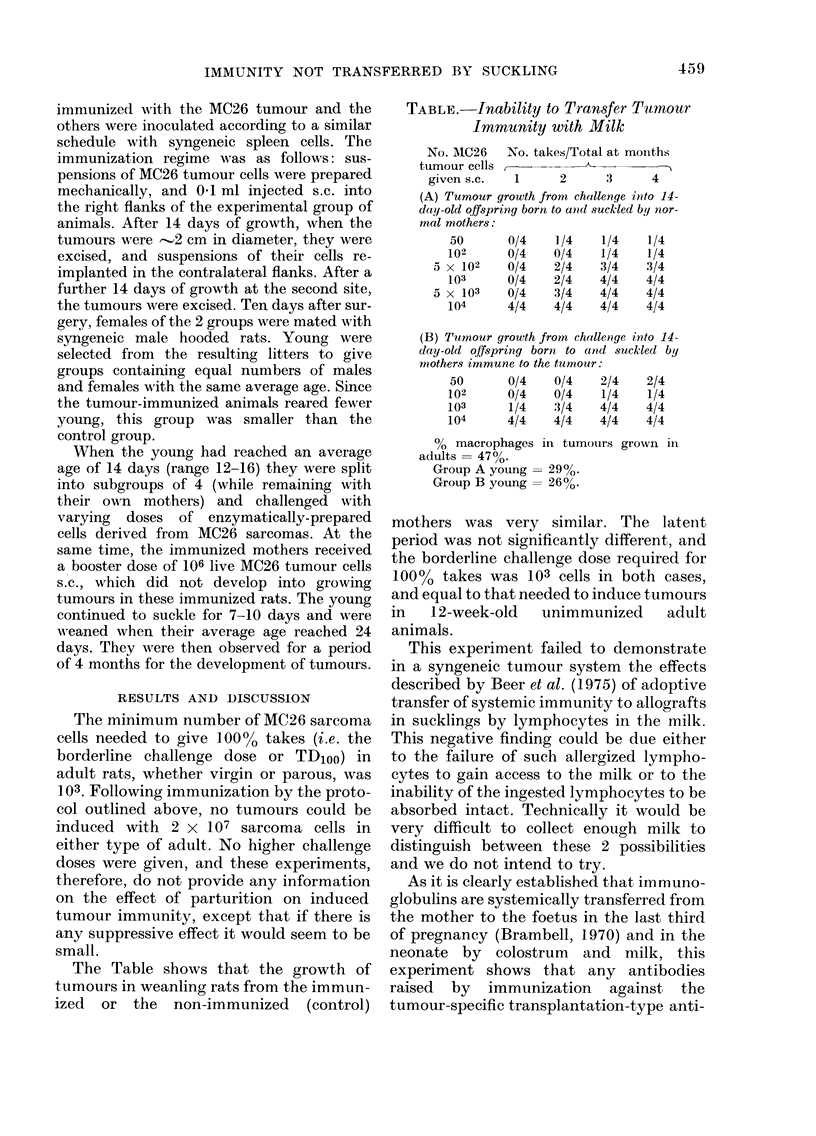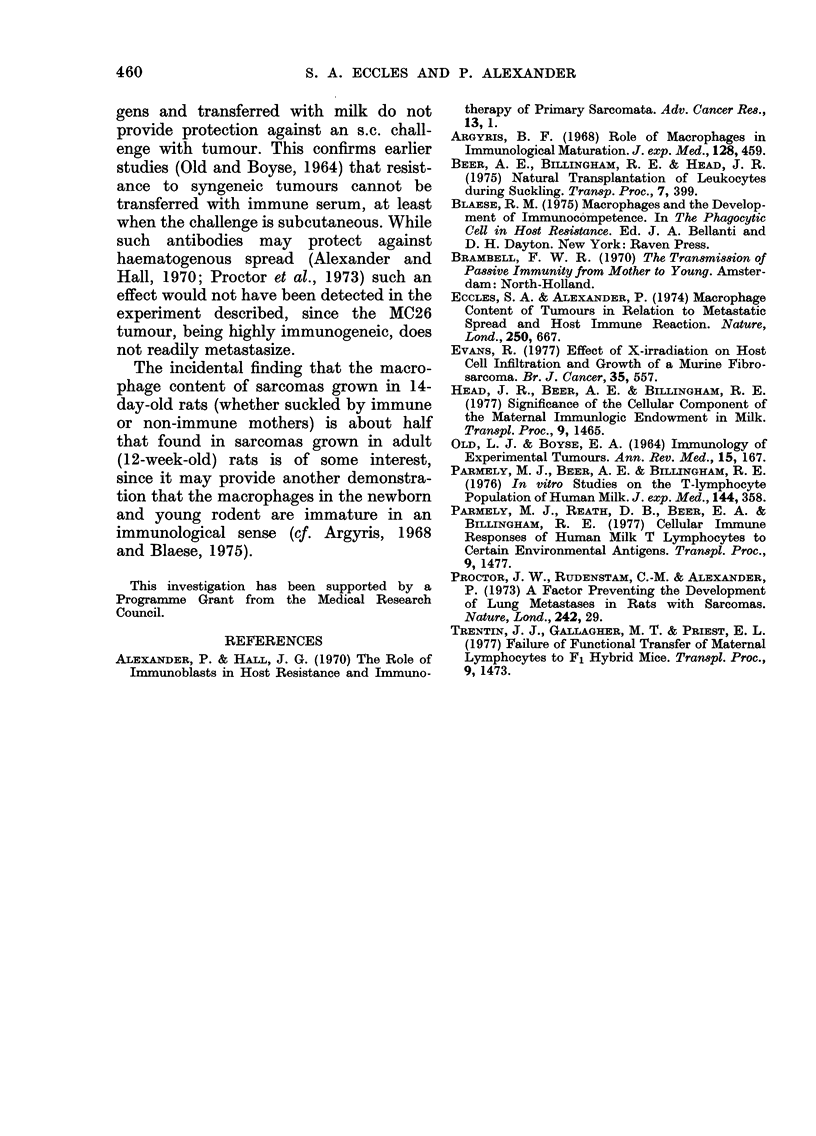# Failure of suckling to transfer immunity to a syngeneic rat sarcoma.

**DOI:** 10.1038/bjc.1978.66

**Published:** 1978-03

**Authors:** S. A. Eccles, P. Alexander

## Abstract

Young born to and suckled by mothers rendered immune to a syngeneic sarcoma showed no acquired resistance to challenge with tumour. Whilst other workers had found that allograft immunity could be transferred by milk, we conclude (1) that lymphocytes present in the milk cannot transfer immunity to tumour-specific antigens when absorbed by suckling, and (2) that transfer of maternal immunoglobulins either pre- or postnatally also does not protect against s.c. inoculated tumour.


Br. J. Cancer (1978) 37, 458

### FAILURE OF SUCKLING TO TRANSFER IMMUNITY TO A SYNGENEIC RAT SARCOMA

S. A. ECCLES AND P. ALEXANDER

*From the Division of Tumour Immunology, Chester Beatty Research Institute, Sutton, Surrey*

Received 9 November 1977 Accepted 14 November 1977

**Summary.—Young born to and suckled by mothers rendered immune to a syngeneic sarcoma showed no acquired resistance to challenge with tumour. Whilst other workers had found that allograft immunity could be transferred by milk, we conclude (1) that lymphocytes present in the milk cannot transfer immunity to tumour-specific antigens when absorbed by suckling, and (2) that transfer of maternal immunoglobulins either pre- or postnatally also does not protect against s.c. inoculated tumour.**

BILLINGHAM and his colleagues (Beer *et al.*, 1975; Parmely *et al.*, 1976; Head *et al.*, 1977; Parmely *et al.*, 1977) found that in all species studied, including man and rat, viable lymphocytes and macrophages are normal components of colostrum and milk, and are present in concentrations similar to those found in peripheral blood. The lymphocytes were shown to be capable of acting as responder or stimulator cells in mixed lymphocyte cultures. While milk leucocytes probably fulfil an important local protective function within the mammary exosecretion, Beer *et al.* (1975) and Head *et al.* (1977) further reported that milk from allogeneic foster mothers influenced the neonate's subsequent immune responses to skin grafts. These authors hypothesized that ingested lymphocytes become systematized in the recipient because they are able to escape from the lumen of the gastro-intestinal tract, as peptic activity in the neonate only becomes demonstrable 15 days after birth. As yet, the observation of Billingham's group that milk-borne cells gain access to the tissues of the young rat and play a role in its immunological development, and in some circumstances act as a form of adoptive immunity, has not been confirmed using labelled cells, and the inability of Trentin *et al.* (1977) to detect lymphocytes of maternal karyotype at the end of the suckling period does not support this interesting hypothesis.

There is abundant evidence (Old and Boyse, 1964) that rejection of syngeneic chemically-induced sarcoma cells after immunization is a specific process which can be adoptively transferred with immune lymphoid cells. Accordingly, milk from immune mothers might be expected to confer on suckling rats specific resistance to tumours. The present experiment was performed to test whether young rats born of and suckled by mothers that had been rendered resistant by immunization to challenge with a highly immunogenic chemically-induced sarcoma, responded differently from comparable young rats born of mothers not exposed to the tumour.

### MATERIALS AND METHODS

Syngeneic Lister hooded rats bred in the Institute were used in the present experiment. A methylcholanthrene-induced sarcoma, referred to as MC26, in its 9th–12th *in vivo* passage, was used because of its high immunogenicity. The macrophage content of the tumours in the different recipients was determined as previously described (Eccles and Alexander, 1974; Evans, 1977).

Eight-week-old female hooded rats were divided into 2 groups: half were hyperimmunized with the MC26 tumour and the others were inoculated according to a similar schedule with syngeneic spleen cells. The immunization regime was as follows: suspensions of MC26 tumour cells were prepared mechanically, and 0·1 ml injected s.c. into the right flanks of the experimental group of animals. After 14 days of growth, when the tumours were ~2 cm in diameter, they were excised, and suspensions of their cells reimplanted in the contralateral flanks. After a further 14 days of growth at the second site, the tumours were excised. Ten days after surgery, females of the 2 groups were mated with syngeneic male hooded rats. Young were selected from the resulting litters to give groups containing equal numbers of males and females with the same average age. Since the tumour-immunized animals reared fewer young, this group was smaller than the control group.

When the young had reached an average age of 14 days (range 12–16) they were split into subgroups of 4 (while remaining with their own mothers) and challenged with varying doses of enzymatically-prepared cells derived from MC26 sarcomas. At the same time, the immunized mothers received a booster dose of $10^6$ live MC26 tumour cells s.c., which did not develop into growing tumours in these immunized rats. The young continued to suckle for 7–10 days and were weaned when their average age reached 24 days. They were then observed for a period of 4 months for the development of tumours.

### RESULTS AND DISCUSSION

The minimum number of MC26 sarcoma cells needed to give 100% takes (*i.e.* the borderline challenge dose or $TD_{100}$) in adult rats, whether virgin or parous, was $10^3$. Following immunization by the protocol outlined above, no tumours could be induced with $2 \times 10^7$ sarcoma cells in either type of adult. No higher challenge doses were given, and these experiments, therefore, do not provide any information on the effect of parturition on induced tumour immunity, except that if there is any suppressive effect it would seem to be small.

The Table shows that the growth of tumours in weanling rats from the immunized or the non-immunized (control) mothers was very similar. The latent period was not significantly different, and the borderline challenge dose required for 100% takes was $10^3$ cells in both cases, and equal to that needed to induce tumours in 12-week-old unimmunized adult animals.

TABLE.—*Inability to Transfer Tumour Immunity with Milk*

| No. MC26 tumour cells given s.c. | No. takes/Total at months 1 | 2 | 3 | 4 |
|---|---|---|---|---|
| (A) *Tumour growth from challenge into 14-day-old offspring born to and suckled by normal mothers:* | | | | |
| 50 | 0/4 | 1/4 | 1/4 | 1/4 |
| $10^2$ | 0/4 | 0/4 | 1/4 | 1/4 |
| $5 \times 10^2$ | 0/4 | 2/4 | 3/4 | 3/4 |
| $10^3$ | 0/4 | 2/4 | 4/4 | 4/4 |
| $5 \times 10^3$ | 0/4 | 3/4 | 4/4 | 4/4 |
| $10^4$ | 4/4 | 4/4 | 4/4 | 4/4 |
| (B) *Tumour growth from challenge into 14-day-old offspring born to and suckled by mothers immune to the tumour:* | | | | |
| 50 | 0/4 | 0/4 | 2/4 | 2/4 |
| $10^2$ | 0/4 | 0/4 | 1/4 | 1/4 |
| $10^3$ | 1/4 | 3/4 | 4/4 | 4/4 |
| $10^4$ | 4/4 | 4/4 | 4/4 | 4/4 |

% macrophages in tumours grown in adults = 47%.

Group A young = 29%.

Group B young = 26%.

This experiment failed to demonstrate in a syngeneic tumour system the effects described by Beer *et al.* (1975) of adoptive transfer of systemic immunity to allografts in sucklings by lymphocytes in the milk. This negative finding could be due either to the failure of such allergized lymphocytes to gain access to the milk or to the inability of the ingested lymphocytes to be absorbed intact. Technically it would be very difficult to collect enough milk to distinguish between these 2 possibilities and we do not intend to try.

As it is clearly established that immunoglobulins are systemically transferred from the mother to the foetus in the last third of pregnancy (Brambell, 1970) and in the neonate by colostrum and milk, this experiment shows that any antibodies raised by immunization against the tumour-specific transplantation-type antigens and transferred with milk do not provide protection against an s.c. challenge with tumour. This confirms earlier studies (Old and Boyse, 1964) that resistance to syngeneic tumours cannot be transferred with immune serum, at least when the challenge is subcutaneous. While such antibodies may protect against haematogenous spread (Alexander and Hall, 1970; Proctor *et al.*, 1973) such an effect would not have been detected in the experiment described, since the MC26 tumour, being highly immunogeneic, does not readily metastasize.

The incidental finding that the macrophage content of sarcomas grown in 14-day-old rats (whether suckled by immune or non-immune mothers) is about half that found in sarcomas grown in adult (12-week-old) rats is of some interest, since it may provide another demonstration that the macrophages in the newborn and young rodent are immature in an immunological sense (*cf.* Argyris, 1968 and Blaese, 1975).

This investigation has been supported by a Programme Grant from the Medical Research Council.

### REFERENCES

ALEXANDER, P. & HALL, J. G. (1970) The Role of Immunoblasts in Host Resistance and Immunotherapy of Primary Sarcomata. *Adv. Cancer Res.*, **13**, 1.

ARGYRIS, B. F. (1968) Role of Macrophages in Immunological Maturation. *J. exp. Med.*, **128**, 459.

BEER, A. E., BILLINGHAM, R. E. & HEAD, J. R. (1975) Natural Transplantation of Leukocytes during Suckling. *Transp. Proc.*, **7**, 399.

BLAESE, R. M. (1975) Macrophages and the Development of Immunocompetence. In *The Phagocytic Cell in Host Resistance*. Ed. J. A. Bellanti and D. H. Dayton. New York: Raven Press.

BRAMBELL, F. W. R. (1970) *The Transmission of Passive Immunity from Mother to Young*. Amsterdam: North-Holland.

ECCLES, S. A. & ALEXANDER, P. (1974) Macrophage Content of Tumours in Relation to Metastatic Spread and Host Immune Reaction. *Nature, Lond.*, **250**, 667.

EVANS, R. (1977) Effect of X-irradiation on Host Cell Infiltration and Growth of a Murine Fibrosarcoma. *Br. J. Cancer*, **35**, 557.

HEAD, J. R., BEER, A. E. & BILLINGHAM, R. E. (1977) Significance of the Cellular Component of the Maternal Immunlogic Endowment in Milk. *Transpl. Proc.*, **9**, 1465.

OLD, L. J. & BOYSE, E. A. (1964) Immunology of Experimental Tumours. *Ann. Rev. Med.*, **15**, 167.

PARMELY, M. J., BEER, A. E. & BILLINGHAM, R. E. (1976) *In vitro* Studies on the T-lymphocyte Population of Human Milk. *J. exp. Med.*, **144**, 358.

PARMELY, M. J., REATH, D. B., BEER, E. A. & BILLINGHAM, R. E. (1977) Cellular Immune Responses of Human Milk T Lymphocytes to Certain Environmental Antigens. *Transpl. Proc.*, **9**, 1477.

PROCTOR, J. W., RUDENSTAM, C.-M. & ALEXANDER, P. (1973) A Factor Preventing the Development of Lung Metastases in Rats with Sarcomas. *Nature, Lond.*, **242**, 29.

TRENTIN, J. J., GALLAGHER, M. T. & PRIEST, E. L. (1977) Failure of Functional Transfer of Maternal Lymphocytes to $F_1$ Hybrid Mice. *Transpl. Proc.*, **9**, 1473.